# “Let’s Keep Pushing the Envelope”

**DOI:** 10.36660/abc.20220470

**Published:** 2022-08-25

**Authors:** Vitor Coimbra Guerra

**Affiliations:** 1 Sickkids Hospital Department of Pediatrics University of Toronto Ontario Canadá Labatt Family Heart Center Sickkids Hospital, Department of Pediatrics, University of Toronto, Ontario – Canadá

**Keywords:** Cardiopatias Congênitas/complicações, Canal Arterial/anormalidades, Permeabilidade do Canal Arterial/anormalidades, Epidemiologia, Técnicas de Diagnóstico Cardiovascular/tendências

O Canal Arterial foi a primeira cardiopatia congênita a ser tratada cirurgicamente em 1938^1^ e recentemente voltou aos holofotes do cenário da cardiologia pediátrica pela possibilidade de tratamento percutâneo em uma população muito vulnerável, os recém-nascidos prematuros e com baixo peso. Aliás, o tópico canal arterial e prematuridade nunca saiu do radar dos neonatologistas e cardiologistas pediátricos, pois sempre foi um desafio o tratamento e, inúmeros trabalhos randomizados, foram feitos para responder questões quanto a melhor forma de tratar e o impacto na sobrevida.

A prematuridade é um dos maiores desafios da saúde pública mundial e tem aumentado a incidência em todo o mundo. Dados recentes, mostram que a incidência de prematuridade mundialmente foi de 10,6 para cada 100 nascidos vivos. Infelizmente o Brasil está entre os 10 países com maior incidência de partos prematuros (11,2 para cada 100 nascidos).^2^

A incidência de persistência do canal arterial é inversamente proporcional a idade gestacional: quanto mais prematuro (< 24 semanas) e menor o peso, maior a incidência e complicações. Existe um longo debate e inúmeras publicações a respeito e sabe-se que há associação entre a persistência do canal arterial e múltiplas morbidades, incluindo a hemorragia intracraniana, enterocolite necrotizante, retinopatia e broncodisplasia pulmonar. Uma vez que o neonatologista e o cardiologista pediátrico entendem qual o momento adequado e necessário, sempre que possível tentam o fechamento com anti-inflamatórios não esteroides como primeira opção. Infelizmente, o sucesso do tratamento é ainda relativamente baixo, em torno de 70%, ou seja, existe uma parcela grande de prematuros que precisam de outras estratégias para o fechamento, até então feito cirurgicamente.^3^

Essa experiência inicial brasileira tem uma grande importância já de imediato por ter ultrapassado dois grandes testes: a viabilidade técnica e o baixo risco de complicações. Houve 100% de sucesso do procedimento, sem maiores complicações (somente 2 pacientes com estenose leve de ramo esquerdo da artéria pulmonar). É importante contextualizar, usando a experiência mundial para um melhor entendimento e traçar estratégias futuras. Sathanandam et al.^4^ agruparam todas as casuísticas até então publicadas usando o dispositivo Amplatzer Piccolo Occluder: um grupo total de 327 pacientes, obtiveram sucesso em 97%, 8 casos de embolização, 4 casos com obstrução do arco aórtico, 4 casos de obstrução da artéria pulmonar esquerda, insuficiência tricúspide em 4 e 2 óbitos relacionados ao procedimento. Hipoteticamente, se inserimos essa experiência inicial brasileira, haveria uma contribuição positiva para estes desfechos. Outro fator positivo nesta experiência brasileira foi o impacto na evolução dos pacientes (79% dos pacientes puderam sair da ventilação mecânica, que é um dos fatores mais importantes e determinantes da repercussão clínica de um canal arterial.

Pela natureza e desenho deste estudo, Manica et al.^5^ objetivaram somente descrever a experiência, usando diversos centros no país, o que implica em não uniformidade da seleção dos pacientes, diferentes operadores, a falta de seguimento de um protocolo único e outros possíveis fatores que comprometeriam a análise dos resultados. O interessante, que por um lado é uma limitação do estudo, por outro lado tornam ainda mais relevantes estes resultados. Eu citaria como exemplo a idade média na época do procedimento, foi de 38 dias +- 17 dias, o que contribui para uma exposição mais prolongada aos efeitos hemodinâmicos de um *shunt* esquerda-direita significativo. A tendência mundial quando se tem mais certeza da indicação é intervir mais precoce no fechamento do canal arterial.

O debate já existente sobre a indicação, ou o momento ideal de abordagem do canal arterial no prematuro provavelmente vai continuar por mais tempo. Porém, uma das direções que este debate deve seguir está sendo definida justamente pela evolução tecnológica (dispositivos adequados), a qual oferece uma nova opção terapêutica que podemos julgar intuitivamente e por estes resultados, oferecendo menos riscos do que o tratamento cirúrgico. Entretanto, precisamos ainda caminhar mais. Existem, inclusive, outras frentes para serem desbravadas, como a possibilidade do procedimento a beira do leito guiadas pelo ecocardiograma.

Os próximos passos, com certeza inclui um alinhamento com os neonatologistas e partir para estudos robustos, bem desenhados, prospectivos, randomizados comparando os resultados e desfechos com outras formas de tratamento, como o farmacológico. Somente assim poderemos agregar de forma concreta e permanente a opção de tratamento percutâneo para os canais arteriais em recém-nascidos prematuros.

A expressão “Let’s keep pushing the envelope” pode ser aplicada em várias situações em cardiologia pediátrica, pois temos que tentar inovar, extender os nossos limites e muitas vezes ultrapassar as barreiras. Em versão diferente, a cardiologia intervencionista vive situações muito semelhantes a cirurgia cardíaca, nas mudanças de paradigmas nas técnicas cirúrgicas, pois sempre houve alguém ou um grupo para “pushing the envelope”. A diferença é que a indústria/tecnologia precisa oferecer recursos para este processo seguir adiante e que sejam recursos acessíveis através do sistema de saúde público, em um contexto socioeconômico para países como o Brasil.
