# Supplementary material for: “Let’s Keep Pushing the Envelope”
Source: Arq Bras Cardiol. 2022 Aug 25;119(3):468–9. [Article in Portuguese] doi: 10.36660/abc.20220470 (PMC9438543; doi:10.36660/abc.20220470)
Supplement: Translation

# Translation: Let’s Keep Pushing the Envelope

**Authors:** Vitor Coimbra Guerra

**Affiliations:** 1 Canada The Hospital for Sick Children SickKids Learning Institute, Toronto, Ontario – Canada

**Keywords:** Heart Defexts, Congenital/complications, Ductus Arteriosus Patent/abnormalities, Epidemiology, Diagnostic Techniques, Cardiovascular/trends

The Ductus Arteriosus (PDA) was the first congenital heart disease to be treated surgically in 1938^1^ and recently returned to the spotlight of the pediatric cardiology scenario due to the possibility of percutaneous treatment in a very vulnerable population, premature newborns and with low weight. In fact, the topic Arterial Canal and prematurity never went off the radar of Neonatologists and Pediatric Cardiologists, because treatment has always been a challenge and numerous randomized studies have been done to answer questions about the best way to treat and the impact on survival.

Prematurity is one of the world’s greatest public health challenges and has increased incidence worldwide. Recent data show that the incidence of prematurity worldwide was 10.6 per 100 live births. Unfortunately, Brazil is among the 10 countries with the highest incidence of premature births (11.2 per 100 births).^2^

The incidence of the PDA is inversely proportional to gestational age: the more premature (< 24 weeks) and the lower the weight, the higher the incidence and complications. There is a long debate and vast literature about it and it is known that there is an association between the PDA and multiple morbidities, including intracranial hemorrhage, necrotizing enterocolitis, retinopathy and pulmonary bronchodysplasia. Once the neonatologist and the pediatric cardiologist understand the appropriate and necessary time, whenever possible they try to close with non-steroids anti-inflammatory (NSAIDS) as the first option. Unfortunately, the success of the treatment is still relatively low, around 70%, that is, there is a large portion of premature infants who need other strategies for closure, until then done surgically.^3^

This initial Brazilian experience is of great importance yet because it has passed two major tests: technical feasibility and low risk of complications. There was 100% success of the procedure, without major complications (only 2 patients with mild stenosis of the left branch of the pulmonary artery). It is important to contextualize, using the world experience for a better understanding and future strategy. Sathanandam et al.^4^ reviewed all cases published using the device Amplatzer Piccolo Occluder: a total group of 327 patients, were successful in 97%, 8 cases of embolism, 4 cases with Aortic Arch obstruction, 4 cases of obstruction of the left pulmonary art, Tricuspid insufficiency in 4 and 2 deaths related to the procedure. Hypothetically, if we include this initial Brazilian experience, there will be a positive contribution to these outcomes. Another positive factor was the impact on the short outcome of patients (79% of patients were able to leave the ventilation of the disease, which is one of the most important and determining factors of the clinical repercussion of the PDA.

By the nature and design of this study, Manica et al.^5^ objective was only to describe the experience, using several centers in the country, which implies the no uniformity of the patients selection, different operators, the lack of follow-up of a single protocol and other possible factors that would compromise the analysisof the results. The interesting point, which on the one hand is one of the limitation of this study, on the other hand make these results even more relevant. I would mention as an example the age at the time of the procedure was 38 days +- 17 days, which contributes to a more prolonged exposure to the hemodynamic effects of a significant left-right shunt. The world’s trend is to intervein earlier once the clinical indication is made.

The debate will continue about the indication, the ideal time to approach the PDA in premature infants. The direction of this debate has changed. The evolution of the technology (appropriate devices) as safe (or even with less risk) than surgical treatment. However, we need to walk further and longer, maybe at a faster pace. There are also other fronts to be explored, such as the possibility of the bedside procedure guided by the echocardiogram.

The next steps certainly include an alignment with neonatologists and move on to robust, well-designed, prospective, randomized studies comparing the results and outcomes with other forms of treatment, such as the pharmacological. Only in this way can we concretely and permanently aggregate the option of percutaneous treatment for PDA in premature newborns/infants.

The expression “Let’s keep pushing the envelope” can be applied in several situations in pediatric cardiology. We must innovate, extend our boundaries, and overcome barriers. In a slightly different version the interventional cardiology deals with very similar situations than pediatric cardiac surgery: the paradigm shift always requires someone or a group to push the envelope. The difference is that the industry/ technology needs to offer resources for this process to move forward, but making the resources accessible through the public health system, in a socioeconomic context of countries as Brazil.
